# Feasibility and safety of endoscopic resection for duodenal gastrointestinal stromal tumors

**DOI:** 10.1055/a-2655-1439

**Published:** 2025-08-07

**Authors:** Shao-Bin Luo, Zu-Qiang Liu, Li Wang, Yi-Qun Zhang, Ming-Yan Cai, Quan-Lin Li, Ping-Hong Zhou

**Affiliations:** 1 92323 Endoscopy Center and Edoscopy Research Institute, Zhongshan Hospital, Fudan University, Shanghai, 200032, China; 2 92323 Shanghai Collaborative Innovation Center of Endoscopy, Shanghai, 200032, China

**Keywords:** Endoscopy Upper GI Tract, Precancerous conditions & cancerous lesions (displasia and cancer) stomach, Endoscopic resection (ESD, EMRc, ...), Endoscopy Lower GI Tract, Polyps / adenomas / ...

## Abstract

**Background and study aims:**

Endoscopic resection for duodenal gastrointestinal stromal tumors (GISTs) is still considered a great challenge with a high risk of complications. This study aimed to evaluate effectiveness and safety of endoscopic resection for duodenal GIST.

**Patients and methods:**

Between June 2013 and August 2024, we performed a retrospective study of patients with duodenal GISTs who underwent endoscopic resection at Zhongshan Hospital. Data on patient characteristics, clinical outcome, and follow-up were collected.

**Results:**

A total of 73 patients with duodenal GISTs were enrolled, including 31 patients who underwent endoscopic submucosal dissection (ESD) and 42 who underwent endoscopic full-thickness resection (EFTR). Mean lesion size was 1.2 ± 0.5 cm and 1.9 ± 0.9 cm, respectively. En bloc resection rates were 96.8% and 95.2%, respectively. Rates of R0 resection were 45.2% and 42.9%, respectively. Rates of R1 resection were 54.8% and 57.1%, respectively. No patient transferred to open surgery. Postoperative adverse events included delayed bleeding (1 case), delayed perforation (1 case), delayed wall edema (2 cases), hydrothorax (1 case), and retroperitoneal infection (1 case). Mean hospital stays were 4.1 ± 2.8 days and 6.2 ± 4.9 days, respectively. No metastasis or duodenal stenosis were detected during the follow-up period (64.8 ± 43.6 months and 61.3 ± 40.2 months, respectively). Local recurrence occurred in one patient with high recurrence risk at 56 months after EFTR.

**Conclusions:**

ESD and EFTR are safe, minimally invasive treatments for duodenal GISTs. Moreover, the EFTR technique may have advantages of completely resecting lesions originating from the deep muscularis propria layer, particularly lesions with extraluminal growth.

## Introduction

Gastrointestinal stromal tumors (GISTs), originating from interstitial cells of Cajal (ICCs), represent the most prevalent subtype of soft tissue sarcomas 1 . While predominantly occurring in the stomach (60%-65%) and jejunoileal regions (20%-25%), duodenal involvement accounts for 3% to 5% of cases 2 . These neoplasms exhibit variable malignant potential with infrequent nodal metastasis, primarily metastasizing to hepatic and peritoneal sites 3 . Radical excision remains the primary therapeutic approach, although duodenal resections pose unique technical challenges due to anatomical proximity to pancreaticobiliary structures and major vasculature, with associated long-term functional sequelae 4 .

Recent advancements in endoscopic interventions have expanded treatment modalities for GISTs. Compared with conventional surgery, endoscopic resection (ER) demonstrates reduced invasiveness, decreased blood loss, and lower perioperative complication rates 5 . Over the past decade, minimally invasive techniques such as endoscopic submucosal dissection (ESD) have gained prominence for their organ-preserving benefits and cost-effectiveness 6 . However, ESD limitations emerge when managing lesions involving the muscularis propria (MP) or serosal layers, where endoscopic full-thickness resection (EFTR) proves more effective by allowing en bloc resection of transmural or extraluminal tumors 6 .

However, recent research on ER of GISTs is mostly focused on the stomach, and there is still a lack of data on ER of duodenal GISTs 7 . Furthermore, long-term oncological outcomes of endoscopic interventions for duodenal GISTs remain inadequately documented. This study evaluated the efficacy and safety of ER for duodenal GISTs.

## Patients and methods

### Patients

**Fig. 1:**
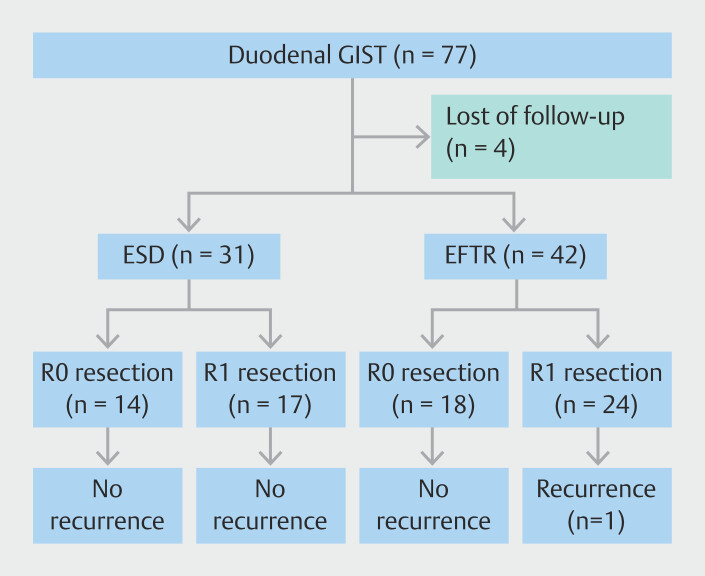
Flowchart of endoscopic resection for duodenal gastrointestinal stromal tumors. EFTR, endoscopic full-thickness resection; ESD, endoscopic submucosal dissection; GIST, gastrointestinal stromal tumor.

Between June 2013 and August 2024, 73 patients with duodenal GIST were treated in our center ( Fig. 1 ). Lesion size and origin were confirmed by endoscopic ultrasonography (EUS) and computed tomography (CT). Inclusion criteria were as follows: 1) the tumor originated in the duodenum; 2) no metastasis detected by EUS or CT; and 3) postoperative pathologically diagnosed as GIST. Exclusion criteria were as follows: 1) evidence of lymph node metastasis or invasion; 2) rich blood supply; and 3) inability to tolerate anesthesia. All patients underwent EFTR and ESD by experienced endoscopists. The research protocol received approval from the committee. Consent forms were obtained from patients.

### Endoscopic procedures

All patients underwent adequate bowel preparation. Treatment was conducted under general anesthesia. The following devices were used as appropriate: a standard endoscope (GIF-Q290J; Olympus), IT knife (KD-611L; Olympus), or Hook knife (KD-620QR; Olympus), clips (HX-600–135; Olympus and Resolution clips). Choice of ER procedure was made by each individual endoscopist, depending on tumor size, location, origin, and growth pattern. ESD is principally suitable for submucosal tumors with a diameter less than 5 cm that originate from the mucosal or submucosal layer. EFTR is mainly suitable for GISTs that originate from deep layers and for which the tumor and the serosal layer cannot be distinguished because they are found to be closely adherent to the serosal layer of extra serosal growth on preoperative EUS and CT evaluation.

**Fig. 2:**
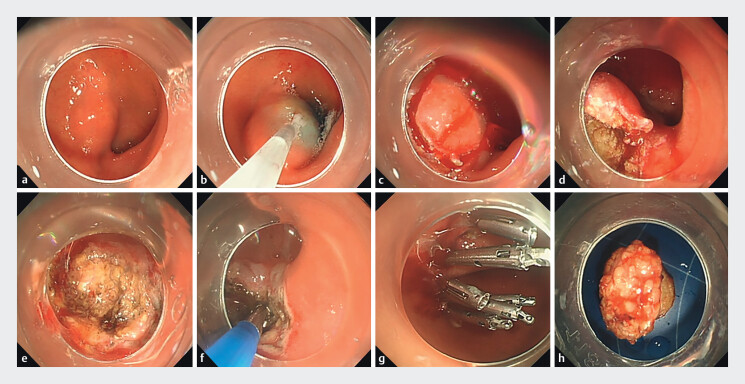
The procedure of ESD for a GIST at the duodenal bulb and descending junction. **a** A submucosal tumor in the bulb-descending junction **b** The submucosal injection. **c,d,e,f** Mucosal incision. **g** The defect was occluded by using metallic clips. **h** Resected en bloc specimen (2.0 cm × 1.5 cm).

The ESD procedure was performed as follows. First, undiluted sodium hyaluronate was injected into the submucosa. Then the initial mucosal incision was made. Dissection was conducted horizontally after adequate separation and depth in the submucosa, and the tumor was resected gradually. The exposed small blood vessels were treated by hemostasis with hot biopsy forceps, and the wound was completely closed with metal clamp. The ESD procedure for duodenal GIST is shown ( Fig. 2 ).

**Fig. 3:**
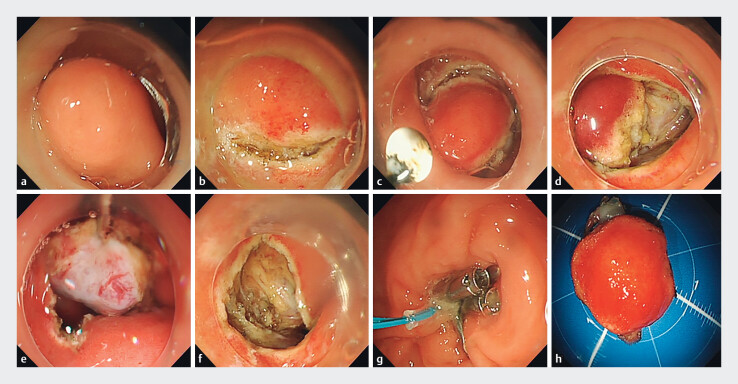
The procedure of EFTR for a GIST at the duodenal bulb. **a** A submucosal tumor at the duodenal bulb. **b,c** After injection into the submucosa, circumferential incision was made as deep as muscularis propria around the lesion. **d,e** Incision into serosal layer around the lesion was performed to create active perforation. **f** The defect in the wall of the duodenum after tumor resection. **g** The defect was occluded by purse-string suture. **h** Resected en bloc specimen (1.8 cm × 1.5 cm).

The EFTR procedure was performed as follows. First, a mixture solution (including 100 mL of normal saline, 1 mL of indigo carmine, and 1 mL of epinephrine) was injected into the submucosa. Then, a circumferential incision was made as deep as MP around the lesion with an IT knife. Incision into the serosal layer around the lesion was performed with a Hook knife to create active perforation. The tumor, including its surrounding MP and serosa, was fetched with a snare. After careful hemostasis, the postresection defect was separately closed with metallic clips, purse-string suture, or OverStitch. A 20-G needle was used to alleviate the intraoperative pneumoperitoneum when needed. Finally, a gastrointestinal decompression tube was placed near the wound for drainage and detection of postoperative hemorrhage. The classic case of EFTR for duodenal GIST is shown in Fig. 3 . Meticulous preservation of the serosal layer was prioritized, when feasible, to prevent pneumoperitoneum development and peritoneal contamination. Post-resection defect management necessitated proficient endoscopic expertise. For defects not exceeding the expanded clip arm span, isolated metallic clip application proved sufficient. Defects exceeding 15 to 20 mm in diameter required combined therapeutic approaches, either through coordinated use of metallic clips with nylon loop or implementation of omental patch reinforcement techniques.

### Evaluation

The mitotic index was calculated by counting 50 consecutive high-power fields (HPFs). Immunohistochemical analysis using antibodies against CD117 (c-kit) and CD34 was performed to confirm presence of GIST. All histopathologic diagnoses were made by three pathologists experienced in gastrointestinal pathology. En bloc resection was defined as excision of the tumor in one piece without piecemeal resection. By definition, R0 resection was a histologically complete en bloc resection with a negative lateral and basal resection margin. R1 resection was defined as removal of all macroscopic disease, with microscopic margins positive for tumor. The Clavien-Dindo classification system was applied to evaluate severity of related adverse events (AEs) 8 . Extraluminal tumors were GISTs with > 50% exophytic growth. Peri-ampullary lesions were those that arise within 2 cm of the duodenal papilla. Technical success was defined as successful ER of the lesion and closure of the incision without conversion to open surgery.

### Postoperative management and follow-up

Patients were kept fasting for 48 hours postoperatively, intravenous antibiotics and parenteral nutrition were administered, and basic vital signs were monitored. Postoperative complications, such as elevated body temperature, hematemesis, chest pain, dyspnea, and abdominal pain, were monitored. Endoscopic surveillance was scheduled at 3, 6, and 12 months postoperatively and once a year after surgery through a gastroscopic check of wound healing and to determine presence of residual or recurrent lesions. CT was recommended once a year.

### Statistical analysis

Statistical analyses were conducted using SPSS Statistics 22.0. Continuous variables were expressed as mean±SD and compared using Student's *t* -test. Categorical variables were analyzed by Fisher's exact test as appropriate. Continuously distributed variables were compared using Student’s *t* -test. Non-continuously distributed variables were compared using Mann-Whitney U test. *P* < 0.05 was considered to indicate statistical significance.

## Results

### Characteristics of patients and lesions

Clinical features of patients and tumors are shown in Table 1 . Thirty-one patients underwent ESD and 42 underwent EFTR. Mean age of patients was 46.2 ± 18.4 years and 47.7 ± 14.6 years, respectively. Among them, 19 patients and 28 patients had no obvious symptoms, respectively. Lesion size was 1.2 ± 0.5 cm and 1.9 ± 0.9 cm, respectively. In the ESD group, 19 lesions were detected in the duodenal bulb, five cases in the bulb-descending junction, six cases in the descending part, and one case was peri-ampullary. In the EFTR group, 25 lesions were located in the bulb, seven cases in the bulb-descending junction, eight cases in the descending part, and two cases were peri-ampullary. The tumor growth pattern was as follows: intraluminal growth (31 cases and 34 cases, respectively) and extraluminal growth (8 cases in the EFTR group). Depth of infiltration was into the submucosa and MP in 29 cases (93.6%) and two cases (6.4%), respectively. All lesions originated from the MP layer in the EFTR group. There were 27 cases (87.1%) with mitotic count < 5/50 HPFs and 4 cases (12.9%) with mitotic count ≥ 5/50 HPFs in the ESD group.

**Table:** **Table 1** Clinical characteristics of duodenal GISTs.

**Characteristics**	**ESD (n = 31)**	**EFTR (n = 42)**	***P* value **
Age, years	46.2 ± 18.4	47.7 ±14.6	0.707
Male, n (%)	18 (58.1%)	22 (52.4%)	0.627
Concomitance chronic diseases, n (%)	2 (6.5%)	3 (7.1%)	1.000
Anticoagulant drugs, n (%)	0 (0%)	1 (2.4%)	-
Symptoms, n (%)			
No symptoms	19 (61.3%)	28 (66.7%)	0.474
Epigastric discomfort	7 (22.6%)	9 (21.4%)	0.905
Abdominal pain	4 (12.9%)	3 (7.1%)	0.436
Hemorrhage	1 (3.2%)	2 (4.8%)	1.000
Lesion size, cm	1.2±0.5	1.9±0.9	0.062
Location of lesion, n (%)			
Bulb	19 (61.3%)	25 (59.5%)	0.878
Bulb-descending junction	5 (16.1%)	7 (16.7%)	1.000
Descending part	6 (19.3%)	8 (19.0%)	1.000
Peri-ampullary	1 (3.2%)	2 (4.8%)	1.000
Tumor growth pattern, n%			0.003
Intraluminal growth	31 (100%)	34 (80.9%)	
Extraluminal growth	0 (0%)	8 (19.1%)	
Infiltration depth, n (%)			< 0.001
Submucosa	29 (93.6%)	0 (0%)	
Muscularis propria	2 (6.4%)	42 (100%)	
Morphology, n (%)			0.693
Regular	28 (90.3%)	39 (92.9%)	
Irregular	3 (9.7%)	3 (7.1%)	
Mucosa, n (%)			0.406
Smooth	30 (96.8%)	38 (90.5%)	
Anabrotic or ulcerative	1 (3.2%)	4 (9.5%)	
Lymph nodes metastasis, n (%)	0 (0%)	0 (0%)	-
Distant metastasis, n (%)	0 (0%)	0 (0%)	-
Mitotic count, HPF, n%			0.459
≤ 5/50	27 (87.1%)	39 (92.9%)	
> 5/50	4 (12.9%)	3 (7.1%)	
NIH risk classification, n%			0.659
Low	20 (64.5%)	26 (61.9%)	
Intermediate	9 (29.0%)	13 (31.0%)	
High	2 (6.5%)	3 (7.1%)	
CD117, n%	31 (100%)	42 (100%)	1.000
CD34, n%	31 (100%)	42 (100%)	1.000
ESD, endoscopic submucosal dissection; EFTR, endoscopic full-thickness resection; GIST, gastrointestinal stromal tumor; HPF, high-power field; NIH, National Institutes of Health.			

### Clinical outcomes and follow-up

Table 2 summarizes clinical outcomes and follow-up. En bloc resection was achieved in 30 patients (96.8%) and 40 patients (95.2%), respectively. R0 resection was achieved in 14 patients (45.2%) and 18 patients (42.9%), respectively. The ESD group had a shorter procedure duration (32.6 ± 19.8 min vs. 64.2 ± 35.7 min, *P* < 0.001) compared with the EFTR group. None of the patients required conversion to open surgery. Gastric tube placement was performed in 11 patients and 19 patients in the ESD and EFTR groups, respectively.

**Table:** **Table 2** Procedure-related characteristics and follow-up.

	**ESD (n = 31)**	**EFTR (n = 42)**	***P* value **
Technical success, n (%)	31 (100%)	42 (100%)	1.000
En bloc resection, n (%)	30 (96.8%)	40 (95.2%)	1.000
R-Status, n (%)			0.845
R0	14 (45.2%)	18 (42.9%)	
R1	17 (54.8%)	24 (57.1%)	
Tumor rupture, n%	0 (0%)	0 (0%)	-
Procedure duration, median (range), min	32.6±19.8	64.2±35.7	< 0.001
Wound closure, n (%)			< 0.001
Metal clips	29 (93.6%)	8 (19.0%)	
Purse-string suture	2 (6.4%)	34 (81.0%)	
Gastric tube, n (%)	11 (35.5%)	19 (45.2%)	0.393
Transferred to open surgery, n%	0 (0%)	0 (0%)	-
Postoperative AEs, n (%)	1 (3.2%)	6 (12.0%)	0.242
Delayed bleeding	0 (0%)	1 (2.4%)	-
Delayed perforation	0 (0%)	1 (2.4%)	-
Duodenal wall edema	1 (3.2%)	1 (2.4%)	-
Hydrothorax	0 (0%)	1 (2.4%)	-
Retroperitoneal infection	0 (0%)	1 (2.4%)	-
Hospital stay, days	4.1 ± 2.8	6.2 ± 4.9	0.015
Adjuvant Imatinib, n%	0 (0%)	1 (2.4%)	-
Follow-up duration, months	64.8 ± 43.6	61.3 ± 40.2	0.718
Local recurrence, n (%)	0 (0%)	1 (2.4%)	-
Metastasis, n (%)	0 (0%)	0 (0%)	-
Stenosis, n (%)	0 (0%)	0 (0%)	-
AE, adverse event; EFTR, endoscopic full-thickness resection; ESD, endoscopic submucosal dissection.			

After the procedure, delayed bleeding occurred in one patient ( Table 3 ). This complication was treated with blood transfusion, endoscopic irrigation to remove the clot, hot biopsy forceps, cauterization of the metal clamp, and gastric tube decompression. After EFTR, delayed perforation accompanied by hydrothorax and retroperitoneal infection occurred in one patient with 20-mm extraluminal growth of lesions in the descending part. This complication was treated with open surgery, blood transfusion, and a chest tube for drainage. Delayed wall edema occurred in three patients, all of whom were treated with nasojejunal tube placement.

**Table:** **Table 3** Details of patients with major postoperative adverse events.

**Case**	**Sex**	**Age**	**Size (mm)**	**Location**	**Growth pattern**	**Layer involved**	**En bloc resection**	**Closure technique**	**Procedure**	**Adverse events**	**POD (days)**	**Clavien- Dindo**	**Therapy**	**Hospital stay (days)**
1	F	46	20	Descending part	Extraluminal growth	Muscularis propria	Yes	Purse-string suture	EFTR	Delayed perforation; hydrothorax; retroperitoneal infection	1	IV	Open surgery; blood transfusion; chest tube for drainage;	53
2	M	57	16	Peri-ampullary	Intraluminal growth	Muscularis propria	Yes	Metal clips	EFTR	Delayed wall edema	1	III	Nasojejunal tube placement	11
3	M	67	20	Bulb	Intraluminal growth	Submucosa	Yes	Metal clips	ESD	Delayed wall edema	2	III	Nasojejunal tube placement	7
4	F	63	28	Bulb	Intraluminal growth	Muscularis propria	Yes	Purse-string suture	EFTR	Delayed bleeding	2	III	Endoscopic hemostasias	9
F, female; M, male. ESD, endoscopic submucosal dissection; EFTR, endoscopic full-thickness resection; POD, postoperative day.														

### Follow-up

**Fig. 4:**
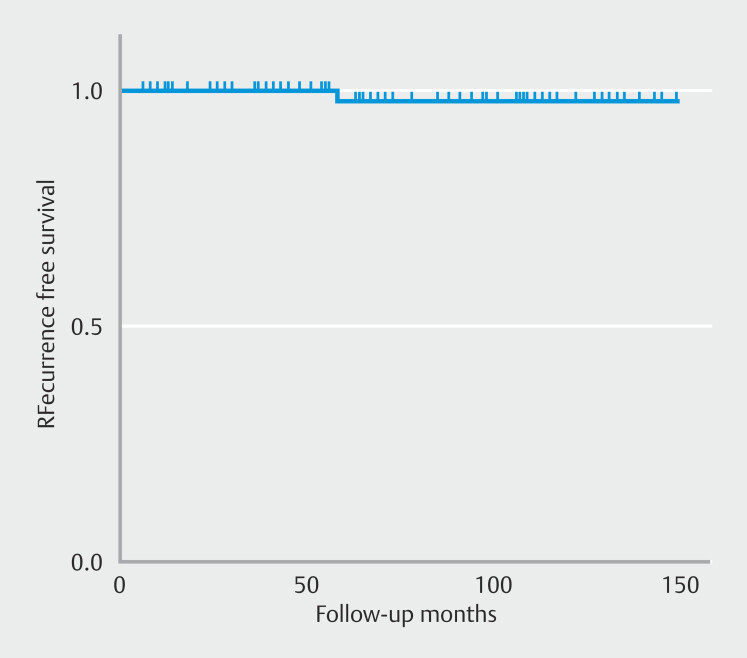
Kaplan-Meier graph of the recurrence-free survival rate.

Follow-up was performed 6 months after the initial procedure to assess outcome. No metastases or stenoses were detected during follow-up (mean duration 64.8 ± 43.6 months and 61.3 ± 40.2 months, respectively). During the follow-up period, one patient had a recurrence at 56 months after EFTR. The recurrent case in the EFTR group was a 2.5-cm GIST that was resected and classified as high risk because of the high mitotic index (29/50 HPF). The Kaplan-Meier plot in Fig. 4 graphically depicts time to recurrence.

## Discussion

Submucosal tumors (SMTs) are frequently identified incidentally during standard upper gastrointestinal endoscopic examinations 8 . These lesions may develop within any layer of the intestinal wall and fall under the category of nonepithelial mesenchymal neoplasms 9 . Unlike other tumor types, SMTs seldom exhibit lymphatic spread, with GISTs constituting the predominant subtype among these submucosal growths 10 . Previous studies indicate that GISTs generally have small dimensions and subtle clinical presentations 11 .

At present, surgical resection is still the common choice for treating duodenal GISTs, including pancreaticoduodenectomy and segmental duodenectomy 12 . However, due to the large surgical wound, high incidence of postoperative complications, and poor quality of life of postoperative patients, the overall effect of surgical resection on duodenal GISTs is not ideal 13 . With advancements in endoscopic technology and its widespread adoption, an increasing number of endoscopists are managing duodenal lesions endoscopically. ER has emerged as a minimally invasive, cost-effective, and diagnostically valuable approach for subepithelial lesions including GISTs, with a favorable safety profile and rapid postoperative recovery 12 . ER of duodenal lesions, especially subepithelial lesions, is still considered a challenging procedure due to the unique anatomical and endoscopic features of the duodenum. The duodenal lumen is rather narrow, and the initial part (bulbar to descending part) is an anti-c-shaped loop, which makes endoscopic operations difficult 14 . Submucosal injection frequently fails to achieve adequate mucosal lifting due to the rich vascularity and Brunner’s glands within the duodenal submucosa 15 . Traditionally, the duodenum has been regarded as a forbidden zone for endoscopic excision of duodenal subepithelial lesions, especially for EFTR 16 . Despite rapid advancements in endoscopic techniques and devices, given the low incidence of duodenal GISTs and technical challenges of ER, few studies have compared long-term survival outcomes of ER and surgical resection in these patients.

In National Comprehensive Cancer Network (NCCN) guidelines, the estimated metastasis rate of GISTs less than 2 cm with high mitosis count (> 5 mitosis/50 HPFs) is approximately 4.6% 17 . Given the technical difficulties of surgical resection, especially after tumors grow larger during surveillance, we suggested that ER be routinely recommended to patients for GISTs less than 2 cm, with consideration of other factors such as their age, comorbidities, and willingness to undergo this treatment. Our treatment approach for all lesions was based on multiple factors: First, duodenal GISTs demonstrate significantly higher malignant potential compared with gastric counterparts. Second, our cohort's mean tumor size (1.9 ± 0.9 cm) exceeded the 1-cm threshold for which current guidelines recommend intervention.

Several studies have shown that ESD and EFTR are safe, feasible, and oncologically equivalent to laparoscopic surgeries 17 . A previous study demonstrated that perforation and bleeding were inherent risks in endoscopic treatment of some GISTs, which may be related to surgeon skill level, intraoperative treatment of blood vessels, and endoscopic suture techniques 18 . In our study, delayed bleeding occurred in only one patient who underwent ER, and it was managed timely and successfully without serious consequences. An important technical issue concerning ER of GISTs in the upper gastrointestinal tract is to avoid macroperforation, and our experience showed that one patient with macroperforation required conversion to surgery. Similarly, in a recently released study, 6.5% and 1.9% of patients in the endoscopic group with duodenal GISTS (≤ 5 cm) encountered delayed bleeding and perforation, respectively 14 . With development of endoscopic techniques, endoscopic suture techniques have made great progress in recent years. Incidence of delayed bleeding and perforation has decreased, and these AEs all were managed timely and successfully, underscoring the critical role of postoperative monitoring and early detection of potential AEs 19 .

Compared with other segments of the gastrointestinal tract, the duodenal wall exhibits significantly underdeveloped muscular tissue, making it more susceptible to iatrogenic rupture during endoscopic interventions 20 . Furthermore, presence of corrosive secretions such as bile and pancreatic enzymes can exacerbate tissue damage at the injury site, potentially leading to secondary perforation 21 . Surgical protocols emphasize the importance of preserving the integrity of the duodenal MP and serosal layers during perforation management 22 . Nevertheless, when pathological lesions demonstrate direct adherence to the MP or serosal structures of the duodenum, complete avoidance of perforation becomes clinically unachievable 23 .

Choice of endoscopic treatment should be based on the specific situation of the tumor, such as the original site, size, and whether it is growing inside or outside the cavity, as well as the clinical experience of the operator. ESD is mainly suitable for small lesions with superficial invasion, whereas EFTR is mainly suitable for GISTs originating from the deep muscularis propria that are growing outside the cavity. ESD and EFTR are mainly performed for lesions located in the first and second portions of the duodenum, whereas it is not suitable for those located in the third and fourth portions of the duodenum.

A major concern in endoscopic management of duodenal GISTs is risk of positive resection margins 24 . Current guidelines recommend complete tumor excision (R0 resection) as the gold standard for localized GISTs 25 . Notably, our data have revealed comparable overall survival outcomes between R0 and R1 resections when excluding cases with intraoperative tumor disruption. This evidence supports considering microscopically margin-positive resection (R1) as a viable alternative for low-risk lesions in anatomically challenging locations when achieving clear margins proves technically demanding. In our cohort, the R0 resection rate was 43.8% (32/73), contrasting with a 56.2% incidence of R1 resections (41/73), consistent with previous reports of suboptimal margin clearance during ER 7 12 . This elevated R1 incidence primarily stems from technical constraints of endoscopic enucleation, in which dissection planes typically approximate the tumor pseudocapsule with minimal inclusion of adjacent normal tissue 26 . Despite frequent microscopic margin involvement, our follow-up data (mean 61.3 months) revealed only one recurrence (1.4%), underscoring the procedure's clinical efficacy.

In our cohort, the recurrent case in the EFTR group was a 2.5-cm GIST that was resected and classified as high risk because of the high mitotic index (29/50 HPF). We recommended that the patient undergo an additional surgical procedure. The patient refused the additional surgery because of the trauma involved and potential for significant postoperative AE and then received imatinib therapy. Current clinical guidelines recognize elevated mitotic activity (≥ 10/50 HPFs) as a significant prognostic indicator for malignant GISTs, correlating with reduced 5-year disease-free survival and overall survival rates 27 . The guideline for selection of patients for adjuvant therapy varies among experts, mainly because no criteria have yet been established for predicting which patients are at high risk of recurrence after removal of primary GISTs. Pathologists have used some clinical and pathological parameters, classified into two gross spread parameters including liver metastasis and peritoneal dissemination; five microscopic spread parameters including lymph node metastasis, vascular, fat, nerve, and mucosal infiltration; and five histological parameters including mitotic count > 10/50 HPF, MP infiltration, coagulative necrosis, perivascular pattern, and severe nuclear atypia, to classify the stage and grade of GISTs, which strongly correlate with prognosis. Our data suggest that complete surgical excision may mitigate the aggressive potential of small GISTs despite high mitotic indices. Presently, preoperative risk stratification for GISTs remains clinically challenging with noninvasive diagnostic modalities 28 . Conservative surveillance strategies risk accelerated tumor progression in lesions with substantial mitotic activity, potentially altering clinical outcomes 29 . These observations underscore the therapeutic imperative for complete resection of GISTs, including lesions < 2 cm 29 . This study further demonstrates favorable long-term outcomes with endoscopic management of small duodenal GISTs that exhibit high-grade histology, preserving optimal quality of life without adjuvant therapy.

Another controversial issue was tumor spillage in EFTR. Tumor disruption, including intraoperative fragmentation, biopsy-related seeding, visceral perforation, or peritoneal contamination, constitutes a critical determinant of GIST recurrence 24 . In our study, all lesions underwent endoscopic resection without tumor disruption. Procedural perforations related to EFTR require differentiation from true tumor rupture, which explicitly excludes iatrogenic mucosal defects per current classification standards 30 . Our data confirm that controlled endoscopic perforation does not elevate peritoneal metastasis risk when the surgeon adheres to standardized rupture definitions. Technical challenges emerge when managing lesions > 5 cm, particularly in moderate-to-high risk scenarios, in which conventional surgical approaches or hybrid laparoscopic-endoscopic techniques may offer superior oncological control. Existing literature indicates that ER for gastric GISTs remains primarily applicable to neoplasms measuring ≤ 50 mm, whereas duodenal counterparts lack substantial research evidence 26 . Technical constraints arise when managing larger lesions endoscopically, particularly regarding radical resection feasibility. Notably, the anatomical complexity of duodenal GISTs suggests that endoscopic management may demonstrate optimal efficacy in smaller lesions 31 .

This study had several limitations. First, the number of cases in this preliminary research was limited because GISTs are rare among the population. Second, potential bias may be inherent in this retrospective study. Hence, additional studies are anticipated in the future.

## Conclusions

In conclusion, our results indicate that ER for duodenal GIST is effective and safe with fairly long follow-up outcomes. The EFTR technique has advantages of completely resecting lesions originating from the deep MP layer, particularly those with extraluminal growth patterns. In future, prospective multicenter studies are needed to further evaluate the efficacy, safety, and long-term outcomes of ER for duodenal GISTs.
